# Supplementary material for: Aging-associated increase of GATA4 levels in articular cartilage is linked to impaired regenerative capacity of chondrocytes and osteoarthritis
Source: eLife. 2026 Aug 12;14:RP106224. doi: 10.7554/eLife.106224 (PMC13466541; doi:10.7554/eLife.106224)
Supplement: Peer review history

# Editor report: eLife Assessment

**DOI:** 10.7554/eLife.106224.4.sa0

**Authors:** Jie Shen

**Affiliations:** Washington University School of Medicine United States

**Keywords:** Compelling, Important

This study presents an **important** finding on the role of GATA4 in aging- and OA-associated cartilage pathology. The conclusions are well supported by **compelling** in vitro and in vivo evidence. This work will be of broad interest to both cell biologists and orthopedic/skeletal health clinicians.

# Referee report: Reviewer #1 (Public review)

**DOI:** 10.7554/eLife.106224.4.sa1

Summary:

This manuscript assesses the differences between young and aged chondrocytes. Through transcriptomic analysis and further assessments in chondrocytes, GATA4 was found to be increased in aged chondrocyte donors compared to young. Subsequent mechanistic analysis with lentiviral vectors, siRNAs, and a small molecule were used to study the role of GATA4 in young and old chondrocytes. Lastly, an in vivo study was used to assess the effect of GATA4 expression on osteoarthritis progression in a DMM mouse model.

Strengths:

This work linked the over expression of GATA4 to NF-kB signaling pathway activation, alterations to the TGF-b signaling pathway, and found that GATA4 increased the progression of OA compared to the DMM control group. Indicating that GATA4 contributes to the onset and progression of OA in aged individuals.

Comments on revised version:

Great work! All my concerns have been well addressed.

# Referee report: Reviewer #2 (Public review)

**DOI:** 10.7554/eLife.106224.4.sa2

Summary:

This study elucidated the impact of GATA4 on aging- and injury-induced cartilage degradation and osteoarthritis (OA) progression, based on the team's finding that GATA expression is positively correlated with aging in human chondrocytes. By integrating cell culture of human chondrocytes, gene manipulation tools (siRNA, lentivirus), biological/biochemical analyses and murine models of post-traumatic OA, the team found that increasing GATA4 levels reduced anabolism and increased catabolism of chondrocytes from young donors, likely through upregulation of the BMP pathway, and that this impact is not correlated with TGF-β stimulation. Conversely, silencing GATA4 by siRNA attenuated catabolism and elevated aggrecan/collagen II biosynthesis of chondrocytes from old donors. The physiological relevance of GATA4 was further validated by the accelerated OA progression observed in lentivirus-infected mice in the DMM model.

Strengths:

This is a highly significant and innovative study that provides new molecular insights into cartilage homeostasis and pathology in the context of aging and disease. The experiments were performed in a comprehensive and rigorous manner. The data were interpreted thoroughly in the context of the current literature.

Weaknesses:

The only aspect that would benefit from further clarification is a more detailed discussion of aging-associated ECM changes in the context of prior literature.

# Referee report: Reviewer #3 (Public review)

**DOI:** 10.7554/eLife.106224.4.sa3

Summary:

This is an exciting, comprehensive paper that demonstrates the role of GATA4 on OA-like changes in chondrocytes. The authors present elegant reverse translational experiments that justify this mechanism and demonstrate the sufficiency of GATA4 in a mouse model of osteoarthritis (DMM), where GATA4 drove cartilage degeneration and pain in a manner that was significantly worse than DMM alone. This could pave the way for new therapies for OA that account for both structural changes and pain.

Strengths:

(1) GATA4 was identified from human chondrocytes.

(2) IHC and sequencing confirmed GATA4 presence.

(3) Activation of SMADs is clearly shown in vitro with GATA4 overexpression.

(4) The role of GATA4 was functionally assessed in vivo using the mouse DMM model, where the authors uncovered that GATA4 worsens OA structure and hyperalgesia in male mice.

(5) It is interesting that GATA4 is largely known to be found in cardiac cells and to have a role in cardiac repair, metabolism, and inflammation, among other things listed by the authors in the discussion (in liver, lung, pancreas). What could this new knowledge of GATA4 mean for OA as a potentially systemically mediated disease, where cardiac disease and metabolic syndrome are often co-morbid?

Weaknesses:

I do not have further comments. Thank you for addressing the previously mentioned concerns.

# Author comment: Author response

**DOI:** 10.7554/eLife.106224.4.sa4

**Authors:** Meagan Makarczyk, Yiqian Zhang, Olivia Bartholomew, Sophie Hines, Suyash Sinkar, Silvia Liu, Craig Duvall, Hang Lin

**Affiliations:** University of Pittsburgh Pittsburgh United States; University of Pittsburgh Pittsburgh United States; University of Pittsburgh Pittsburgh United States; University of Pittsburgh Pittsburgh United States; University of Pittsburgh Pittsburgh United States; University of Pittsburgh Pittsburgh United States; Vanderbilt University Nashville United States; University of Pittsburgh Pittsburgh United States

The following is the authors’ response to the previous reviews

**Reviewer #2 (Public review):**

The only aspect that would benefit from further clarification is a more detailed discussion of aging-associated ECM changes in the context of prior literature.

Thank you. Please refer to the new section (Lines 604-617)

**Reviewer #3 (Public review):**

(1) It would be useful to explain why GATA4 was chosen over HIF1a, which was the most differentially expressed.

Thank you. Please refer to Lines 530-537.

“Of note, Hypoxia-Inducible Factor 1α (HIF1 α) was the most differentially expressed gene predicted to regulate chondrocyte aging. The connection between HIF1 α and aging has been previously reported.[32] Furthermore, additional studies have investigated HIF1α in association with OA and assessed its use as a therapeutic target.[33,34] Therefore, we decided to focus on GATA4, which was less studied in chondrocytes but highly associated with cellular senescence, an aging hallmark. However, our selection did not dampen the importance of HIF1α and other molecules listed in Figure 1D in chondrocyte aging. They can be further studied in the future using the same strategy employed in the current work.”

(2) In Figure 5, it would be useful to demonstrate the non-surgical or naive limbs to help contextualize OARSI scores and knee hyperalgesia changes.

In the current study, we focused on the DMM control and DMM Gata4 virus groups so we did not include a sham control group. We recognized this was a limitation of this study.

(3) While there appear to be GATA4 small-molecule inhibitors in various stages of development that could be used to assess the effects in age-related OA, those experiments are out of scope for the current study.

We agree with this comment that the results are still preliminary, which was the reason that we put it in the supplementary materials. However, we felt like the result is informative, which will support the potential of GATA4 as a therapeutic target and inspire the development of more specific inhibitors. Therefore, we would still keep the results in the current study.
